# Splenic Infarction as a Rare Complication of Acute Brucellosis: A Case Report and Brief Review of Literature

**DOI:** 10.7759/cureus.79627

**Published:** 2025-02-25

**Authors:** Geetanjali Puvvada, Abhijathya Chintha, Hariharan G, Sharada V Kutty

**Affiliations:** 1 General Medicine, All India Institute of Medical Sciences, Mangalagiri, Vijayawada, IND; 2 General Medicine/Pulmonary, Critical Care, and Sleep Medicine, All India Institute of Medical Sciences, Mangalagiri, Vijayawada, IND

**Keywords:** doxycycline, endemic infections, human brucellosis, multidrug therapy, occupational exposure, splenic infarction

## Abstract

Brucellosis, a zoonotic infection caused by *Brucella* species, typically manifests as a febrile illness with systemic involvement. Rare complications, such as splenic infarction, are less commonly encountered. We present a case of a 48-year-old female with prolonged fever and left hypochondrial pain. Diagnostic imaging and blood culture confirmed the diagnosis of brucellosis with splenic infarction. A tailored antibiotic regimen resulted in significant improvement. This case underscores the importance of timely diagnosis and management in endemic regions to prevent severe complications.

## Introduction

Brucellosis, also known as Malta fever or undulant fever, is a zoonotic infection caused by bacteria of the genus *Brucella*. Splenic abscesses and thrombosis are described limitedly in the literature [1-3]. The disease is primarily transmitted to humans through direct contact with infected animals, consumption of unpasteurized dairy products, or inhalation of aerosols [4,5]. Brucellosis, like tuberculosis, presents as a chronic granulomatous disease. Its clinical presentations are highly variable, ranging from asymptomatic infection to severe systemic disease. *Brucella* is a monospecific genus known as *Brucella melitensis*. All other species are subtypes and have an interspecies homology of above 87%. There are various phenotypic differences and host preferences. This is attributed to specific outer membrane protein markers [5,6]. This report presents a rare instance of brucellosis with splenic infarction, aiming to highlight its clinical course, diagnostic considerations, and management strategies.

## Case presentation

A 48-year-old female with a history of rearing cattle presented to our hospital with fever and malaise for approximately 20 days. This was followed by a 10-day history of left hypochondrial pain. The patient reported a pain intensity of 6-7/10, which was partially relieved with analgesics. She denied a history of rash, cough, diarrhea, headache, vomiting, or joint symptoms. She had lost six kilograms of weight in the preceding two months. Her past medical and surgical history was unremarkable.

On clinical examination, she was afebrile, with a pulse rate of 106 beats per minute, blood pressure of 110/70 mmHg, and respiratory rate of 21 breaths per minute. Bilaterally, upper jugular cervical lymph nodes were palpable with an approximate size of 1 * 1 cm. Abdominal examination revealed tenderness in the left hypochondrium, and no organomegaly was noted on superficial palpation. Deep palpation was not possible due to pain. However, there was no evidence of guarding or rigidity. A note was made of normal bowel sounds.

The laboratory investigations (Table 1) revealed mild anemia, elevated inflammatory markers, and significant liver dysfunction with elevated transaminases, alkaline phosphatase, and bilirubin. Albumin was low, indicating possible chronic illness. Lactate dehydrogenase was elevated, consistent with tissue injury or infarction. Total leucocyte count and platelets were within normal limits, but relative neutrophilia (90%) suggested an ongoing inflammatory or infectious process. Renal function was within normal limits.

**Table 1:** Laboratory investigations.

Laboratory investigations	Result	Reference range
White blood cell count	5.7 × 10⁹/L	4.0–11.0 × 10⁹/L
Hemoglobin	10.0 g/dL	12.0–16.0 g/dL (female); 13.0–17.0 g/dL (male)
Platelet count	150 × 10⁹/L	150–450 × 10⁹/L
Erythrocyte sedimentation rate (ESR)	18 mm/h	<20 mm/h (male); <30 mm/h (female)
C-reactive protein (CRP)	19.6 mg/L	<5 mg/L
Alanine transaminase (ALT)	296 U/L	7–56 U/L
Aspartate transaminase (AST)	369 U/L	10–40 U/L
Alkaline phosphatase (ALP)	475 U/L	44–147 U/L
Total bilirubin	2.5 mg/dL	0.1–1.2 mg/dL
Albumin	28 g/L	35–50 g/L
Creatinine	1.0 mg/dL	0.7–1.3 mg/dL (male); 0.6–1.1 mg/dL (female)
Lactate dehydrogenase (LDH)	456 U/L	140–280 U/L

**Figure 1:**
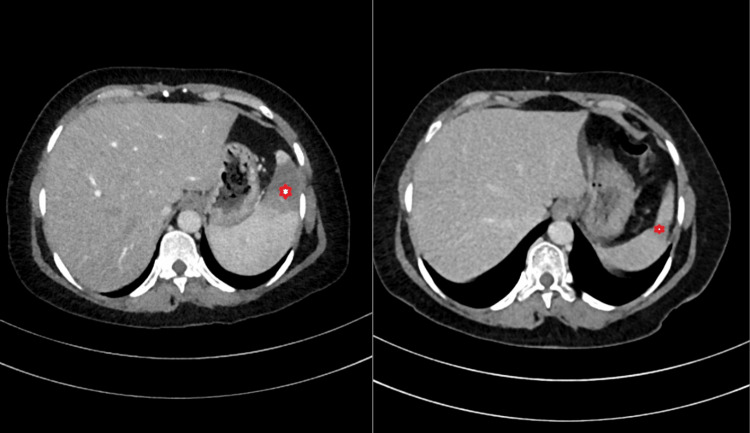
Contrast-enhanced CT of the abdomen. Left panel: Anterior splenic infarct pre-treatment. Right panel: Resolution of the splenic infarct after six weeks of appropriate antibiotic therapy.

**Figure 2:**
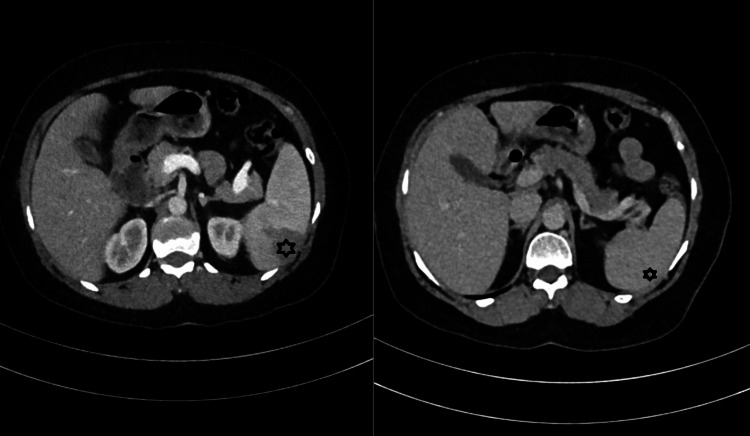
Contrast-enhanced CT of the abdomen. Left panel: Posterior splenic infarct pre-treatment. Right panel: Resolution of the splenic infarct after six weeks of appropriate antibiotic therapy.

Imaging showed a grade 1 fatty liver, splenomegaly with hypoechoic lesions (suggesting abscesses or tuberculomas), and wedge-shaped splenic infarcts on computed tomography (Figures 1, 2). Chest X-ray was normal, and transesophageal echocardiography did not reveal vegetations.

Bacteria were isolated from an automated blood culture after three days of incubation. Gram staining revealed gram-negative coccobacilli. Further analysis using matrix-assisted laser desorption ionization-time of flight (MALDI-TOF) mass spectrometry (MS) identified the organism as *Brucella*. The specific species was not identified, as the database for rare organisms in MALDI-TOF MS did not include this species. However, since treatment does not vary by *Brucella* species, the sample was directly sent for antibiotic susceptibility testing after identification as *Brucella*. Sensitivity to doxycycline, tetracycline, and trimethoprim/sulfamethoxazole was noted.

Treatment was initiated with tablet doxycycline 100 mg twice daily and tablet rifampicin 600 mg once daily and the patient was discharged with out-patient follow-up. Upon follow-up after six weeks, the patient’s general condition improved, fever did not recur, and abdominal pain subsided. The patient tolerated the treatment well. The relative neutrophilia, hyperbilirubinemia, and transaminitis resolved on follow-up.

## Discussion

Brucellosis remains a global public health concern, particularly in endemic regions [7]. It is primarily transmitted through the ingestion of contaminated dairy products or contact with infected animals [8]. Splenic infarction in brucellosis is exceedingly rare and often underdiagnosed due to nonspecific symptoms [7,9]. The pathogenesis of splenic infarction in brucellosis includes direct vascular involvement or immune-mediated vasculitis [8-10]. Early recognition requires a high index of suspicion, especially in patients from endemic areas presenting with persistent fever and left upper quadrant pain. The differential diagnoses include embolic events (e.g., atrial fibrillation), hematological conditions (e.g., sickle cell anemia), and infective endocarditis. Diagnostic confirmation typically relies on a combination of serology, blood cultures, and imaging studies [11]. Contrast-enhanced computed tomography (CECT) of the abdomen is particularly useful for identifying splenic infarcts. Prolonged therapy is often required, particularly in cases involving complications like splenic infarction.

Our patient was a 48-year-old female with occupational exposure to cattle who presented with fever, malaise, and pain in the left hypochondrium. This clinical presentation aligns closely with previously reported cases in the literature, where fever, abdominal pain, and systemic symptoms were prominent in *Brucella*-associated splenic infarction. A notable symptom in the current case is weight loss (six kilograms over two months), also reported in cases by Wang et al. (2017) [12] and Shi et al. (2023) [13]. The patient was afebrile at presentation but had tachycardia (pulse rate of 106 bpm) and palpable upper jugular cervical lymph nodes, consistent with systemic inflammatory response and possible lymphatic involvement.

The laboratory results indicated mild (hemoglobin = 10.0 g/dL), elevated inflammatory markers (CRP = 19.6 mg/L), and significant liver dysfunction (alanine transaminase = 296 U/L, aspartate transaminase = 369 U/L, alkaline phosphatase = 475 U/L, bilirubin = 2.5 mg/dL), consistent with systemic involvement. Similar findings of elevated transaminases and inflammatory markers were noted in cases by Dursun et al. (2012) and Shi et al. (2023). The presence of elevated lactate dehydrogenase (456 U/L) in our case points toward a possibility of splenic infarction, as seen in Alkan et al. (2022) and Hachfi et al. (2012). Notably, the total leucocyte count and platelets were within normal limits, a feature observed in most reported cases, further pointing to chronic inflammation rather than acute infection. Imaging revealed a grade 1 fatty liver, splenomegaly, and wedge-shaped splenic infarcts. The splenic lesions are comparable to those in cases by Salgado et al. (2002) and Wang et al. (2017), where splenic infarcts were confirmed on imaging [12-17].

Blood cultures in the current case confirmed *Brucella* species, sensitive to doxycycline, tetracycline, and trimethoprim/sulfamethoxazole. Treatment with doxycycline and rifampicin was initiated and continued for six weeks. This regimen mirrors the treatment strategies reported in cases like Dursun et al. (2012) [16] and Alawad et al. (2022) [18], emphasizing doxycycline-based regimens. The patient’s outpatient follow-up aligns with reports of prolonged treatment courses, such as six weeks in Ucmak et al. (2014) [19] and two months in Lee et al. (2010) [20]. The literature indicates that splenic infarcts generally resolve or reduce in size over time with appropriate treatment, as seen in Shi et al. (2023) (six weeks) [13] and Alkan et al. (2022) (12 weeks) [14]. The same has been noted in our case.

The current case findings closely parallel those in the literature, particularly regarding clinical presentation, laboratory findings, imaging, and treatment outcomes. This case reports a lesser-known complication, which is important to countries where *Brucella* is endemic. In this report, we wish to emphasize the importance of early diagnosis and tailored treatment in *Brucella*-associated splenic infarction. We also wish to emphasize the importance of a holistic amalgam of clinical, radiological, and microbiological methods in the diagnosis of infectious diseases.

## Conclusions

This case highlights one of the rarer presentations of brucellosis. We endorse a detailed investigation into occupational exposures to avoid missing key differential diagnoses. The clinical presentation, laboratory findings, and imaging features emphasize the diagnostic challenges and the need for a comprehensive evaluation of all patients. The resolution of symptoms with timely therapy underscores the importance of early diagnosis and individualized management for optimal patient outcomes.
